# Endothelial Exostosin‐1 Is Needed for Glycocalyx Integrity and Inflammatory Resolution in Acute Lung Injury

**DOI:** 10.1096/fj.202601250R

**Published:** 2026-10-01

**Authors:** Yuki Kawasaki, Hiroyuki Tomita, Kodai Suzuki, Chihiro Takada, Hirotaka Asano, Soichiro Kano, Yugo Wakayama, Hirotsugu Fukuda, Toru Minamiyama, Genki Yoshimura, Ryo Kamidani, Yoshinori Kakino, Tomotaka Miura, Yuichiro Kitagawa, Tetsuya Fukuta, Takahito Miyake, Akio Suzuki, Kazu Matsumoto, Nobuyuki Tetsuka, Shozo Yoshida, Yoichi Maekawa, Yu Yamaguchi, Hideshi Okada

**Affiliations:** ^1^ Department of Emergency and Disaster Medicine Gifu University Graduate School of Medicine Gifu Japan; ^2^ Department of Tumor Pathology Gifu University Graduate School of Medicine Gifu Japan; ^3^ Center for One Medicine Innovative Translational Research Gifu University Institute for Advanced Study Gifu Japan; ^4^ Department of Infection Control Gifu University Graduate School of Medicine Gifu Japan; ^5^ Department of Internal Medicine Gifu Seiryu Hospital Gifu Japan; ^6^ Department of Pharmacy Gifu University Hospital Gifu Japan; ^7^ Department of Abuse Prevention Emergency Medicine Gifu University Graduate School of Medicine Gifu Japan; ^8^ Department of Parasitology and Infectious Diseases Gifu University Graduate School of Medicine Gifu Japan; ^9^ Division of Preemptive Food Research Preemptive Food Research Center (PFRC), Gifu University Institute for Advanced Study (GUiAS) Gifu Japan; ^10^ Division of Animal Medical Science Center for One Medicine Innovative Translational Research (COMIT), Gifu University Institute for Advanced Study (GUiAS) Gifu Japan; ^11^ Human Genetics Program Sanford Burnham Prebys Medical Discovery Institute La Jolla California USA

**Keywords:** endothelial cells, exostosin‐1, glycocalyx, inflammation, microcirculation

## Abstract

The endothelial glycocalyx depends on Exostosin‐1 (Ext1)‐mediated heparan sulfate synthesis; however, its endothelial cell‐autonomous role in acute inflammation remains unclear. Therefore, we examined the role of Ext1 during acute inflammation. In this study, endothelial‐specific Ext1‐deficient mice (Ext1^flox/flox^ and VE‐cadherin‐Cre) were generated, and their response to endotoxin challenge was investigated using transcriptomics and imaging. Transcriptomic profiling revealed downregulation of heparan sulfate‐ and glycocalyx‐related genes, with upregulation of genes associated with immune activation. Fluorescence imaging demonstrated lectin‐ and heparan sulfate‐defined glycocalyx depletion; dextran extravasation confirmed impaired barrier integrity. Ultrastructural analyses demonstrated a near‐complete absence of the luminal glycocalyx at baseline in Ext1‐deficient lungs. Following lipopolysaccharide administration, Ext1‐deficient mice exhibited altered cytokine kinetics, delayed clearance of neutrophils and CD11b^+^ myeloid cells, increased late‐phase macrophage accumulation, and prolonged pulmonary edema, indicating impaired resolution of inflammation. Gene set enrichment analysis revealed upregulated leukocyte transendothelial migration, chemokine signaling, and vascular endothelial growth factor pathways, consistent with a persistently activated endothelial phenotype. Endothelial Ext1 is indispensable for heparan sulfate biosynthesis, endothelial glycocalyx integrity, and timely inflammation resolution. Loss of Ext1 leads to an intrinsically pro‐inflammatory endothelial state characterized by impaired heparan sulfate–dependent signaling and dysregulated leukocyte–endothelial interactions, heightened vascular permeability, and prolonged tissue injury. Our findings support glycocalyx‐targeted therapy in sepsis, acute respiratory distress syndrome, diabetes‐associated inflammation, and other conditions of endothelial vulnerability.

## Graphical abstract

Endothelial Ext1 is essential for maintaining heparan sulfate–rich endothelial glycocalyx integrity. Loss of Ext1 reduces glycocalyx thickness, resulting in enhanced leukocyte adhesion, increased vascular permeability, and exaggerated inflammatory responses following lipopolysaccharide (LPS) challenge. These changes lead to delayed inflammatory resolution and prolonged lung injury.

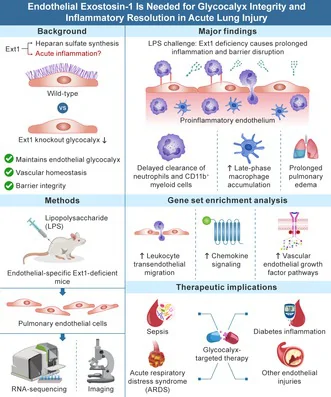

## 1 Introduction

Organ injury begins and resolves at the vascular interface. The vascular endothelial glycocalyx (eGCX) covers the luminal surface of healthy endothelial cells and plays a pivotal role in microvascular and endothelial physiology. It regulates vascular permeability, mechanotransduction, leukocyte trafficking, and the intravascular distribution of circulating inflammatory mediators [1, 2, 3, 4, 5, 6, 7, 8]. Its major components include proteoglycans with glycosaminoglycan side chains, among which heparan sulfate (HS) is the most abundant [9, 10, 11]. HS biosynthesis requires the exostosin family of glycosyltransferases, particularly Exostosin‐1 (Ext1), which catalyzes HS chain polymerization [12]. Complete loss of Ext1 abolishes HS synthesis and results in embryonic lethality, underscoring its essential biological role [13, 14].

eGCX degradation has been reported in sepsis, diabetes, acute kidney injury, cardiovascular disease, and other systemic conditions, and is associated with organ dysfunction and worse clinical outcomes [15, 16, 17, 18, 19, 20, 21]. We previously showed that diabetic mice exhibit reduced pulmonary Ext1 expression, impaired eGCX integrity, and delayed inflammatory resolution [22], suggesting that compromised HS biosynthesis may prolong inflammation. However, whether endothelial‐specific Ext1 directly governs the resolution of inflammation has remained unclear. Despite extensive studies on organ‐level injury in sepsis and acute lung inflammation, the vasculature has often been overlooked as an active driver of disease progression. The vascular endothelium forms a dynamic interface that regulates leukocyte recruitment, cytokine distribution, and tissue homeostasis [23]. Given this central role, even subtle alterations in endothelial HS can exert disproportionately large effects on inflammatory propagation and vascular dysfunction. Conventional models (systemic Ext1 deletion, heparinase‐mediated HS degradation, or diabetes) cannot isolate endothelial‐intrinsic HS functions, because HS is synthesized by many cell types [24].

To address this gap, we established a vascular endothelium‐specific Ext1 conditional knockout model, enabling selective disruption of endothelial HS biosynthesis while preserving systemic HS expression. This model provides a unique tool to define how endothelial HS regulates glycocalyx integrity, vascular permeability, inflammatory responses, and lung injury progression as well as resolution. We hypothesized that endothelial Ext1, by sustaining HS‐dependent glycocalyx structure, is required to promote the resolution of acute inflammation. To test this, mice with endothelial cell‐specific deletion were generated and challenged with endotoxin to determine whether endothelial Ext1 deficiency delays inflammation resolution and to characterize associated transcriptomic alterations.

## 2 Methods

### 2.1 Mice

All animal procedures were performed in accordance with the NIH Guide for the Care and Use of Laboratory Animals (8th Edition, 2011) and approved by the Institutional Animal Research Committee of Gifu University (Approval No. 30–190, Gifu, Japan). All efforts were made to minimize animal suffering and to reduce the number of animals used.

Endothelial cell‐specific Ext1 knockout mice were generated using Ext1^flox/flox^ [25]. Vascular endothelial (VE)‐cadherin‐Cre transgenic mice [26] and Rosa26‐CAG‐LSL‐tdTomato reporter mice (B6;129S6‐Gt (ROSA) 26Sortm9 (CAG‐tdTomato) Hze/J; Jackson Laboratory, stock No. 007905) were obtained from Jackson Laboratories (Bar Harbor, ME, USA). Endothelial cell–specific Ext1 knockout mice were generated by crossing VE‐cadherin‐Cre mice with Ext1^flox/flox^ mice. VE‐cadherin‐Cre(−); Ext1^flox/flox^ littermates served as controls. For endothelial fluorescence imaging experiments, VE‐cadherin‐Cre mice were crossed with Rosa26‐CAG‐LSL‐tdTomato reporter mice to visualize endothelial cell boundaries. For the line‐scan imaging comparisons, control mice were VE‐cadherin‐Cre(+); Ext1^+/+^; Rosa26‐tdTomato, and Ext1‐deficient mice were VE‐cadherin‐Cre(+); Ext1^flox/flox^; Rosa26‐tdTomato because tdTomato visualization requires Cre recombinase activity in both groups. Both male and female mice (8–12 weeks old) were used. Animals were housed under controlled conditions (25°C; 12‐h light/dark cycle) with free access to standard chow (CE‐2, CLEA Japan) and tap water.

### 2.2 Isolation of Pulmonary Endothelial Cells

Pulmonary endothelial cells were isolated from three VE‐cadherin‐Cre(−); Ext1^flox/flox^ control mice and three endothelial‐specific Ext1‐deficient (Cre^+^) mice aged 8–12 weeks. Briefly, lungs were enzymatically dissociated using a Lung Dissociation Kit (Miltenyi Biotec, Cat. No. 130–095‐927) to generate single‐cell suspensions, which were filtered through 70‐μm strainers. Leukocytes were removed by negative selection with CD45 MicroBeads, followed by positive selection using CD31 MicroBeads (Miltenyi Biotec, Germany) and magnetic‐activated cell sorting (MS columns, MACS system). Endothelial cell purity was confirmed by flow cytometric analysis of CD31 expression. Purified cells were immediately processed for RNA extraction.

### 2.3 RNA Extraction

Total RNA was isolated from purified lung endothelial cells using the Maxwell RSC simplyRNA Tissue Kit (Promega, Cat. No. AS1340) following the manufacturer's protocol. Samples were homogenized in thioglycerol‐containing lysis buffer with DNase treatment to remove genomic contamination. RNA was eluted in nuclease‐free water and stored at −80°C until analysis.

### 2.4 Transcriptomic Profiling

RNA‐sequencing was used as the primary transcriptomic approach. Microarray analysis was additionally performed in a subset of samples as an independent validation dataset.

### 2.5 Library Preparation for Transcriptome Sequencing

RNA‐seq libraries were prepared using the NEBNext Ultra RNA Library Prep Kit for Illumina (NEB, Cat. No. E7760) with indexing barcodes according to the manufacturer's protocol. Libraries were purified with AMPure XP beads and quality‐controlled on an Agilent Bioanalyzer 2100 system. Paired‐end sequencing (150 bp) was performed on an Illumina NovaSeq 6000 platform.

### 2.6 Pre‐Processing and Read Alignment

Read quality was assessed using FastQC and MultiQC. Adapter trimming was conducted with Trimmomatic [27], and low‐quality bases and poly‐A/T sequences were removed using PRINSEQ‐lite [28]. Clean reads were aligned to the mouse reference genome (GRCm38) using the STAR aligner [29].

### 2.7 Quantification and Differentially Expressed Gene (DEG) Analysis

Gene‐level read counts were obtained using featureCounts from the Subread package [30], and DEG expression was assessed using edgeR [31]. Counts were normalized to counts per million (CPM) and the trimmed mean of M‐values. Genes expressed in fewer than two samples were excluded. Statistical significance was defined at a false discovery rate (FDR) < 0.05.

### 2.8 Principal Component Analysis and Clustering

Principal component analysis was performed using the R prcomp package based on Euclidean distance. Hierarchical clustering was conducted using the amap and gplots packages, including genes expressed at > 1 CPM in at least two samples, applying the complete linkage method.

### 2.9 Enrichment Analysis

Gene Ontology (GO) enrichment was analyzed using the topGO package [32] with Fisher's exact test under the classic method. Reactome pathway enrichment analysis was performed using ReactomePA [33], org.Mm.eg.db annotation data were used.

### 2.10 Gene Set Enrichment Analysis (GSEA)

GSEA was conducted through the Basepair platform (Basepair Inc., New York, NY). Data were processed through the “Expression count (STAR)” pipeline followed by differential expression assessment using DESeq2. Pathway enrichment was tested against the Molecular Signatures Database (MSigDB) using ranked gene lists based on fold change and statistical significance, and significance was determined by permutation testing with FDR correction.

### 2.11 In Vivo Animal Studies

Mice underwent a 16‐h fast before intraperitoneal administration of lipopolysaccharide (LPS; 15 mg/kg; MilliporeSigma) to minimize variability in metabolic status and to standardize experimental conditions. LPS was administered in a total volume of 100 μL per mouse. Sham animals received an equivalent volume of PBS. Animal allocation was performed in a blinded fashion. Survival was monitored every 6 h. For tissue harvest, mice were anesthetized with medetomidine (0.3 mg/kg), midazolam (4 mg/kg), and butorphanol (5 mg/kg) by intraperitoneal injection. Adequate anesthesia was confirmed by the absence of corneal and paw‐withdrawal reflexes. Animals were sacrificed by exsanguination via the buccal artery.

### 2.12 Serum Preparation and Enzyme‐Linked Immunosorbent Assay (ELISA)

Blood samples were collected from the buccal artery, allowed to clot for 2 h at room temperature, and centrifuged (2 000 × g at 4°C for 20 min). Serum IL‐6 concentrations were measured using an ELISA kit (R&D Systems, Cat. No. M6000B) (*n* = 6 mice per group per time point).

### 2.13 In Vivo Permeability Assay

Vascular permeability was assessed using intravenous injection of FITC–dextran (70 kDa) as described previously [34]. Mice were administered FITC‐dextran (60 mg/100 g body weight), and blood was collected retro‐orbitally 4 h later. Serum fluorescence was measured (492/525 nm), and concentrations were calculated from standard curves (*n* = 6 mice per group).

### 2.14 Histopathological Examination

Lungs harvested at 0 or 48 h after LPS injection were fixed in 10% formalin, paraffin‐embedded, and sectioned (4 μm). Hematoxylin–eosin staining was performed. Pulmonary edema and neutrophil infiltration were scored using standardized criteria [35] by blinded observers (*n* = 6 mice per group).

### 2.15 Scoring of Lectin‐Staining Intensity

Glycocalyx integrity was assessed by wheat germ agglutinin (WGA) staining using frozen lung sections. Sections were incubated with biotinylated WGA (1:200) overnight at 4°C and subsequently probed with DyLight‐594 streptavidin (1:200). Fluorescence intensity was quantified using ImageJ across 10 randomly selected non‐overlapping high‐power fields per sample by a blinded investigator (*n* = 6 mice per group).

### 2.16 Quantitative Line‐Scan Fluorescence Analysis

For quantitative assessment of endothelial glycocalyx integrity and vascular permeability, fluorescence line‐scan analysis was performed across pulmonary capillary cross‐sections. Line profiles were acquired perpendicular to the vessel axis to simultaneously capture tdTomato fluorescence (endothelial reference signal; red) and lectin, heparan sulfate, or FITC–dextran signals (green). Peak fluorescence intensities were extracted using ImageJ software.

For lectin and heparan sulfate analyses (Figure 4A,B), green signal intensity was quantified at the spatial position corresponding to the tdTomato peak representing the endothelial cell surface. Values were normalized to the tdTomato peak intensity to generate a green‐to‐red fluorescence ratio.

For vascular permeability assessment using FITC–dextran, regions of interest were defined outside the endothelial boundary where tdTomato signal intensity was minimal or absent, representing the extravascular compartment. Mean extravascular green fluorescence intensity was quantified and normalized to the tdTomato peak intensity within the adjacent vessel, yielding a standardized permeability index. Five pulmonary capillary cross‐sections were analyzed per mouse (*n* = 6 mice per group; total 30 capillaries per group).

### 2.17 Immunohistochemistry and Immunofluorescence

Sections were incubated with primary antibodies against CD11b (Abcam, ab16667; 1:200), F4/80 (Bioss, bs‐10701R; 1:200), or heparan sulfate (10E4 clone; Amsbio, #370255; 1:100) overnight at 4°C.

For CD11b and F4/80 staining, immunoreactivity was detected using the VECTASTAIN Elite ABC system (Vector Laboratories) according to the manufacturer's instructions and visualized with 3,3′‐diaminobenzidine (DAB). Sections were counterstained with hematoxylin.

For heparan sulfate staining, sections were incubated with an Alexa Fluor 488‐conjugated secondary antibody and analyzed by fluorescence microscopy.

Quantitative analyses were performed in six mice per group.

### 2.18 Electron Microscopy

Glycocalyx ultrastructure was evaluated by scanning and transmission electron microscopy as described [36, 37]. Perfusion fixation using a lanthanum nitrate‐containing buffer allowed visualization of the glycocalyx layer. Scanning electron microscopy (SEM) was performed using an S‐4800 microscope (Hitachi); transmission electron microscopy (TEM) was performed using an HT‐7700 system after resin embedding and ultrathin sectioning (80 nm thickness) (*n* = 3 mice per group).

### 2.19 Microarray Analysis

Microarray analysis was performed on whole‐lung tissue harvested 30 h after LPS challenge (*n* = 4 mice per group) using the SurePrint G3 Mouse GE microarray platform (Agilent). Whole‐lung tissue was analyzed to avoid potential transcriptional artifacts introduced by enzymatic dissociation and endothelial cell isolation after LPS exposure.

Differentially expressed genes were defined by fold change ≥ 2 or ≤ 0.5 with *p* < 0.01. Data were analyzed using MeV and DAVID. GSEA was applied for pathway enrichment analysis. All microarray data were deposited in the Gene Expression Omnibus (GEO) under accession number GSE313973.

### 2.20 Data and Statistical Analysis

Data are presented as individual values with the group mean, or as mean ± standard error of the mean, as indicated in the figure legends. Normality was assessed using the Shapiro–Wilk test. Comparisons between two groups were performed using Student's two‐tailed *t*‐test for normally distributed data and the Mann–Whitney U test for non‐normally distributed data. Survival analyses were performed using the log‐rank test. *p* < 0.05 was considered statistically significant. All analyses were performed using GraphPad Prism version 7.02 (GraphPad Software, La Jolla, CA, USA).

## 3 Results

### 3.1 Profile of Endothelial Cell‐Specific Ext1 Knockout Mice

Endothelial cell‐specific Ext1 knockout mice were generated by crossing Ext1^flox/flox^ mice with VE‐cadherin‐Cre transgenic mice (Figure 1A). To validate Cre activity, Ext1^flox/flox^; VE‐cadherin‐Cre mice were further crossed with Rosa26‐LSL‐tdTomato reporter mice, allowing tdTomato fluorescence to identify Cre‐expressing endothelial cells (Figure 1B). Pulmonary endothelial cells isolated from these mice were subjected to transcriptomic analysis. Principal component analysis and hierarchical clustering demonstrated clear segregation between wild‐type and Ext1‐deficient endothelial cells, confirming the biological robustness of the RNA‐seq dataset (Figure 2). Ext1 expression was reduced in Cre^+^ endothelial cells (Figure 1C,D), accompanied by downregulation of multiple HS‐biosynthetic and core protein genes, including those encoding N‐deacetylase/N‐sulfotransferases, HS 6‐O‐sulfotransferases, EXT2, and core proteins of glypicans and syndecans (SDCs) (Figure 1E), indicating globally impaired glycocalyx biosynthesis and assembly following Ext1 deletion.

**FIGURE 1:**
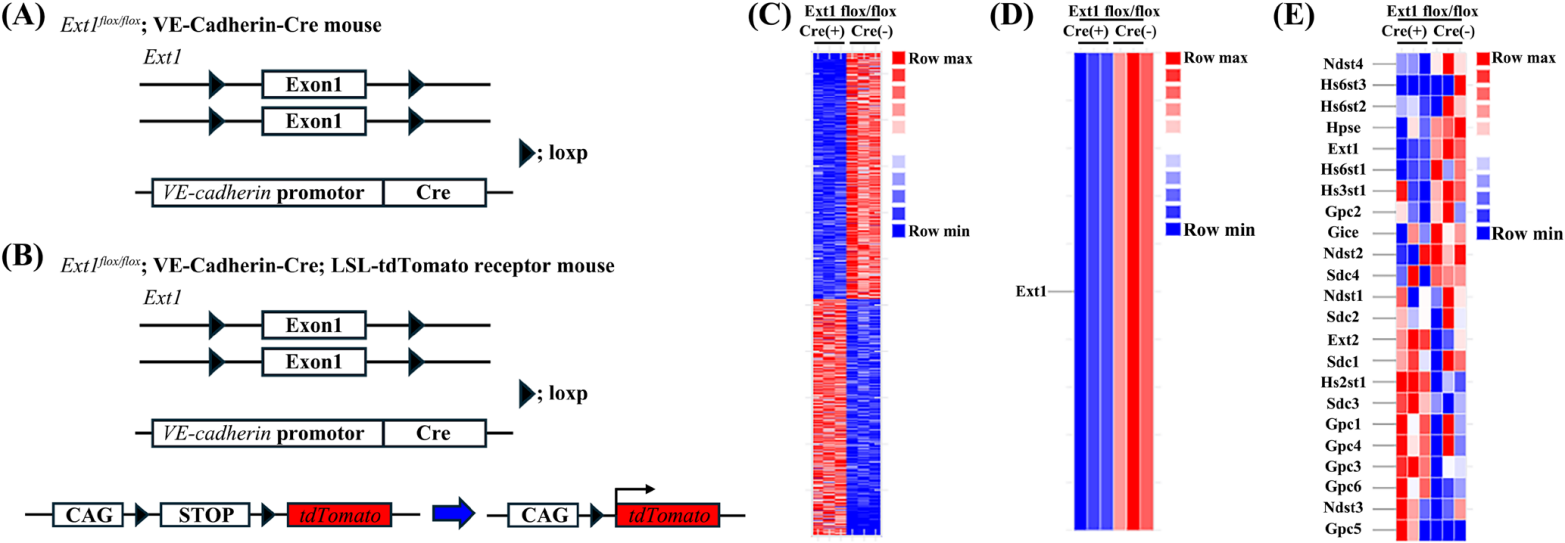
Generation and validation of endothelial cell–specific Ext1 knockout mice. (A) Schematic representation of the Ext1^flox/flox^ allele and the VE‐cadherin‐Cre transgene used to generate endothelial cell‐specific Ext1 knockout mice. (B) Breeding strategy for Ext1^flox/flox^; VE‐cadherin‐Cre; Rosa26‐LSL‐tdTomato reporter mice, in which Cre recombinase activity results in tdTomato expression in endothelial cells. (C) RNA‐sequencing heatmap showing global transcriptomic differences between Ext1‐deficient (Cre^+^) and control (Cre^−^) endothelial cells. (D) Expression levels of Ext1 in Cre^+^ and Cre^−^ endothelial cells, confirming efficient gene deletion. (E) Downregulation of heparan sulfate‐ and glycocalyx biosynthesis‐related genes (*Ndst* family, *Hs6st* isoforms, *Ext2*, glypicans, syndecans) in Ext1‐deficient endothelial cells.

**FIGURE 2:**
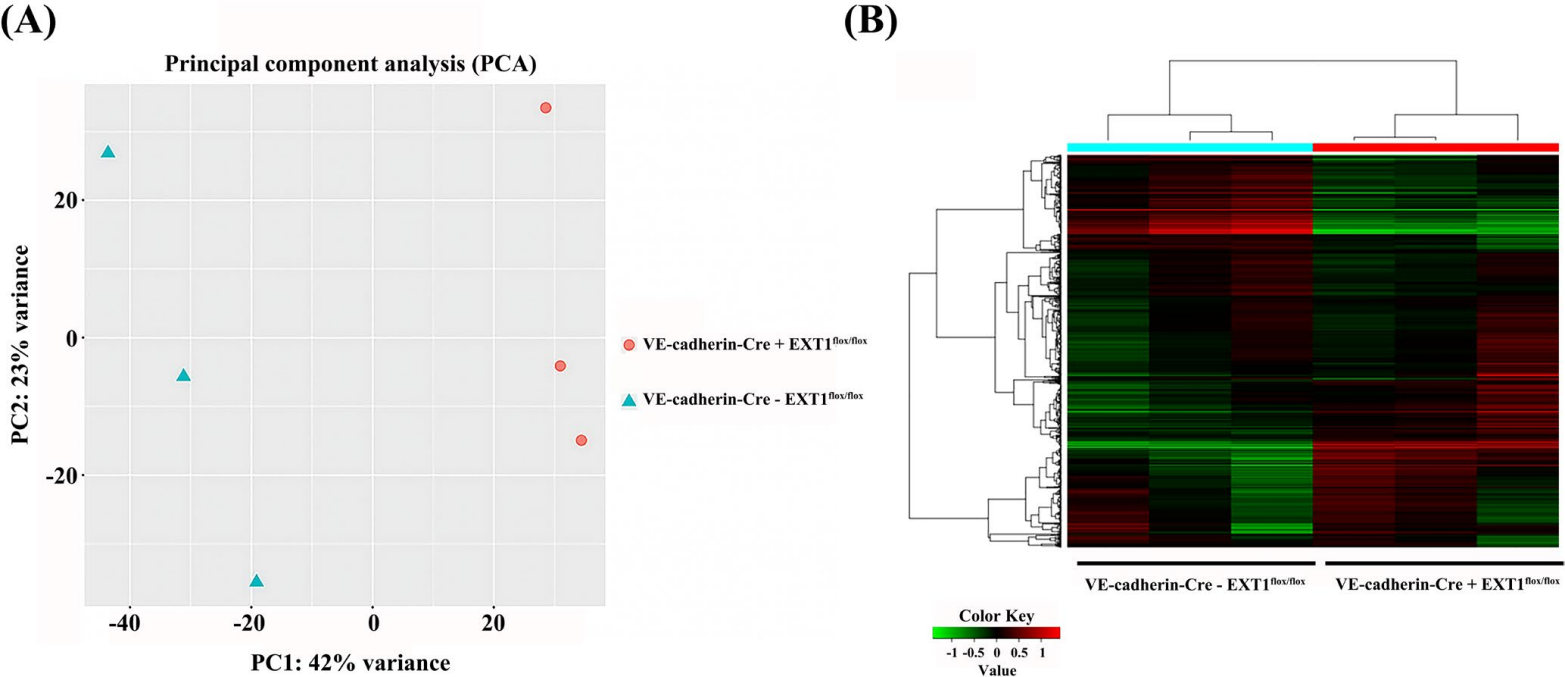
Transcriptomic overview of endothelial Ext1 deficiency. (A) Principal component analysis showing clear separation of Cre^+^ versus Cre^−^ endothelial samples. (B) Hierarchical clustering heatmap confirming reproducible genotype‐specific transcriptional signatures.

Gene ontology enrichment analysis revealed that upregulated genes in Ext1‐deficient endothelial cells were enriched in immune‐related categories such as mononuclear‐cell differentiation, adaptive immune responses, and cytokine‐receptor activity (Figure 3). In contrast, downregulated genes were associated with extracellular matrix organization, cell adhesion, and angiogenesis (Figure 4). These data demonstrate that endothelial Ext1 deficiency induces a shift toward a pro‐inflammatory endothelial transcriptome while suppressing key components of glycocalyx biosynthesis.

**FIGURE 3:**
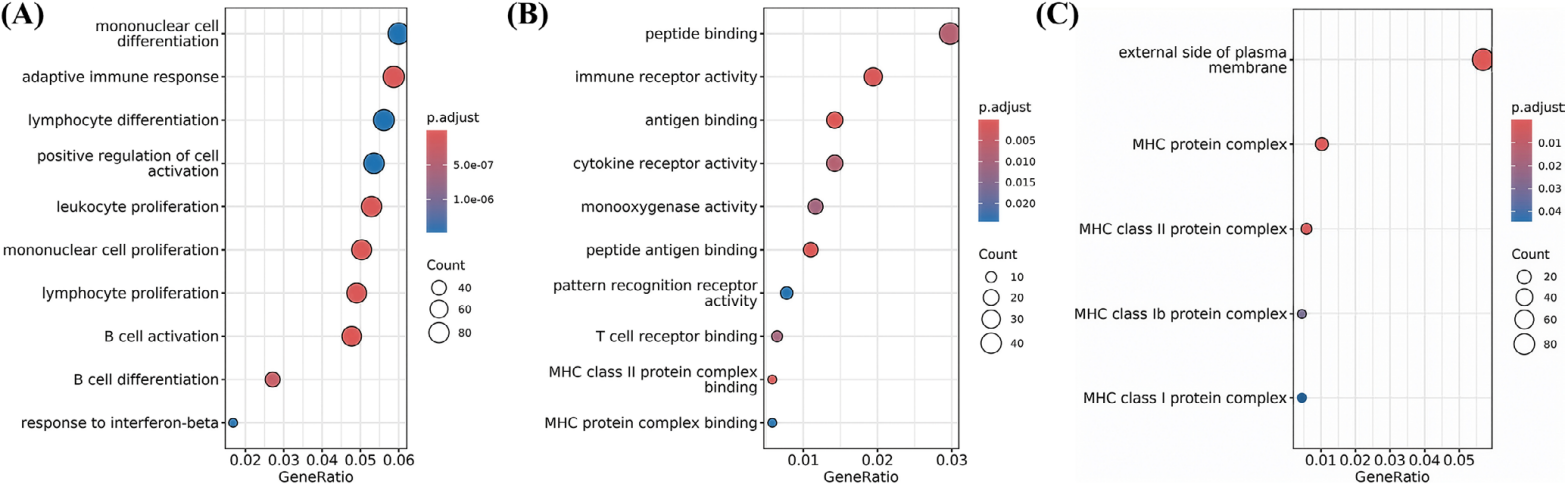
Gene ontology (GO) enrichment analysis of up‐regulated genes in Ext1‐deficient endothelial cells. (A) Enriched biological processes, including mononuclear cell differentiation and adaptive immune responses. (B) Molecular function categories highlighting cytokine receptor activity and antigen‐binding pathways. (C) Cellular component enrichment demonstrating increased expression of immune‐related surface proteins, including myosin heavy chain (MHC) complexes.

**FIGURE 4:**
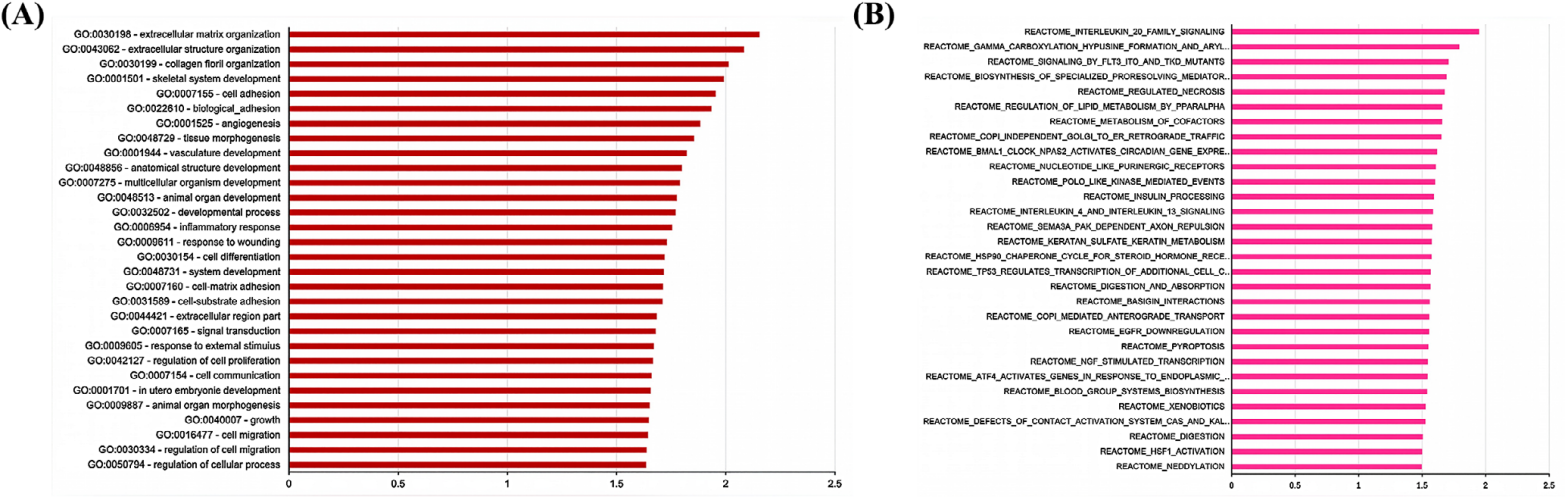
GO enrichment analysis of down‐regulated genes in Ext1‐deficient endothelial cells. (A) Suppression of biological processes related to extracellular matrix organization, angiogenesis, and endothelial adhesion. (B) Reactome pathway analysis demonstrating down‐regulation of extracellular matrix (ECM) remodeling, vascular homeostasis, and structural integrity programs.

### 3.2 Loss of Endothelial Ext1 Impairs Glycocalyx Structure and Endothelial Barrier Integrity

To visualize the eGCX in vivo, VE‐cadherin‐Cre; Ext1^flox/flox^ mice were crossed with Rosa26‐LSL‐tdTomato reporter mice, in which tdTomato fluorescence identifies endothelial cells. In control lungs, lectin staining with wheat germ agglutinin (WGA), which labels the eGCX [38], colocalized with tdTomato‐positive endothelial surfaces, delineating a continuous glycocalyx layer (Figures 5A,B and 6A). In contrast, Ext1‐deficient mice exhibited reduced WGA lectin fluorescence intensity, and the line‐scan profiles showed decreased green peaks coinciding with the red tdTomato signals (Figures 5A,B and 6A), consistent with glycocalyx loss. Similarly, HS immunofluorescence revealed strong staining along tdTomato‐positive endothelial membranes in control lungs, whereas the signal was substantially reduced in Ext1‐deficient mice. The corresponding line‐scan profiles confirmed decreased green HS peaks overlapping with the red tdTomato signals (Figures 5C,D and 6B).

**FIGURE 5:**
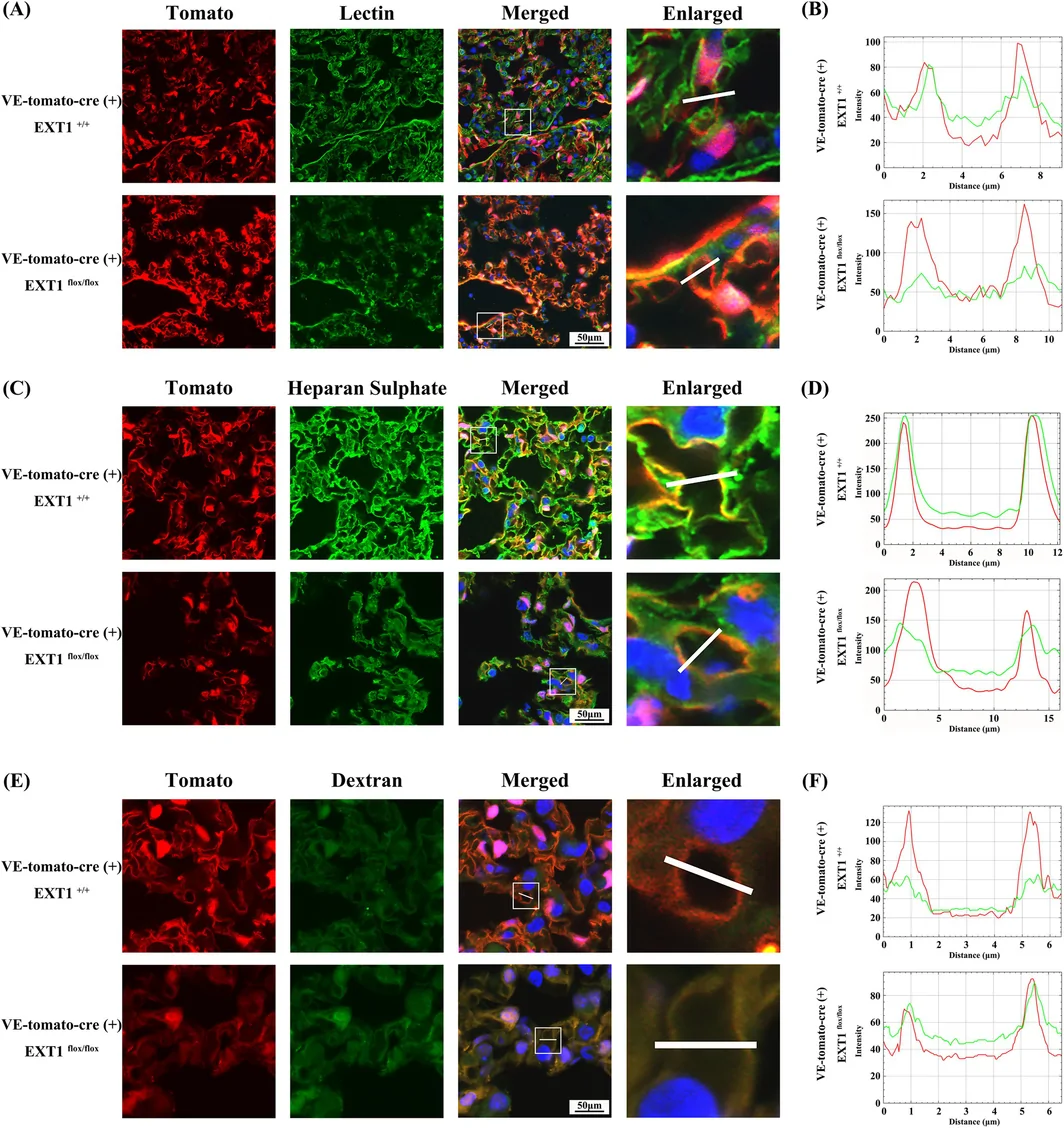
Loss of endothelial glycocalyx in Ext1‐deficient mice demonstrated by fluorescence imaging. (A) Fluorescence microscopy of wheat germ agglutinin (lectin) staining (green) and tdTomato‐labeled endothelial cells (red) in lung tissue. Representative line‐scan locations are indicated by white grids, with enlarged views shown in the right panels. (B) Representative merged line‐scan profile demonstrating reduced lectin signal overlapping the endothelial surface in Cre^+^ mice. (C) Immunofluorescence staining for heparan sulfate (green) showing reduction at tdTomato‐positive endothelial surfaces in Cre^+^ mice. (D) Quantification of lectin and heparan sulfate fluorescence intensity ratios relative to tdTomato signal obtained from line‐scan analyses. (E) Fluorescein isothiocyanate (FITC)‐dextran permeability assessment demonstrating outward dextran fluorescence displacement beyond the endothelial boundary in Cre^+^ mice. (F) Representative line‐scan fluorescence profiles confirming increased vascular leakage. Data are expressed as mean ± standard error of the mean (SEM). *p* < 0.05 versus Cre^−^ controls.

**FIGURE 6:**
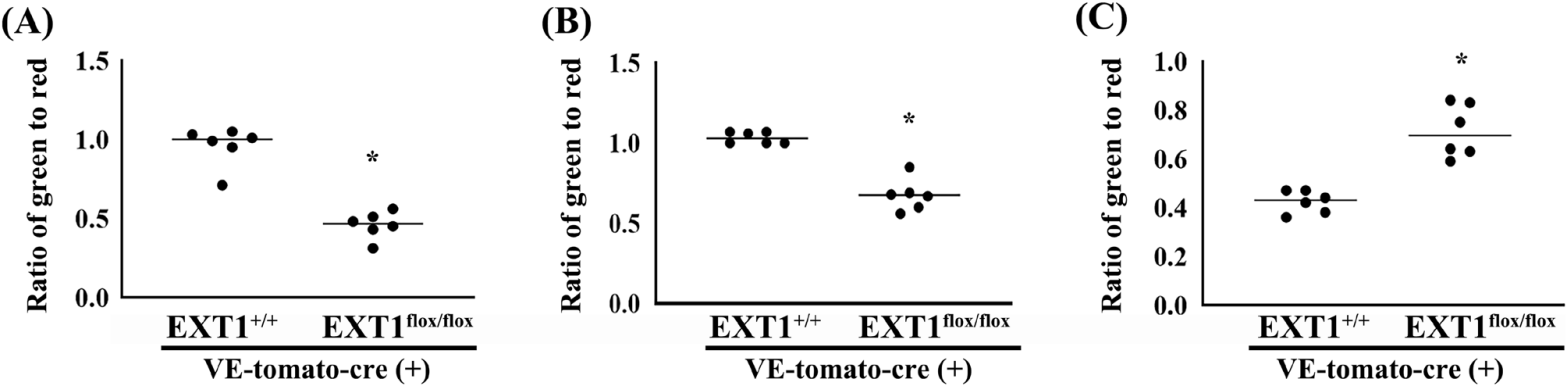
Quantitative fluorescence analysis of endothelial glycocalyx disruption. (A) Ratio of lectin fluorescence (green) to tdTomato endothelial signal (red) measured at the endothelial surface peak position. (B) Ratio of heparan sulfate fluorescence to tdTomato endothelial peak intensity. (C) Ratio of extravascular FITC–dextran fluorescence to the adjacent endothelial tdTomato peak intensity, reflecting vascular permeability. All fluorescence ratios were derived from line‐scan analyses as detailed in the Supplementary Methods. Data are presented as individual values with the group means. Statistical significance was assessed using the Mann–Whitney U test for panels A and B and Student's two‐tailed *t*‐test for panel C. *p < 0.01 versus Cre^−^ controls.

To assess the functional consequence of glycocalyx disruption, fluorescein‐labeled dextran was intravenously injected to evaluate vascular permeability. In control mice, dextran fluorescence was confined within the vascular lumen, whereas the green dextran signal extended beyond the red tdTomato peaks on line‐scan analysis in Ext1‐deficient mice, indicating extravasation and increased vascular permeability (Figures 5E,F and 6C). Collectively, these findings demonstrate that endothelial Ext1 loss leads to pulmonary glycocalyx structural depletion and endothelial barrier integrity impairment.

### 3.3 Baseline Ultrastructural Analysis Reveals Pre‐Existing Glycocalyx Depletion in Ext1‐Deficient Endothelial Cells

To directly assess glycocalyx ultrastructure, SEM and TEM were performed under non‐inflammatory conditions. SEM revealed a dense, granular glycocalyx coating on endothelial luminal surfaces in control mice, whereas Ext1‐deficient endothelial cells displayed sparse, irregular surface projections with loss of glycocalyx continuity (Figure 7A). TEM using lanthanum nitrate staining confirmed a continuous electron‐dense glycocalyx layer lining endothelial surfaces in control lungs, while it was thinned and discontinuous in Ext1‐deficient mice (Figure 7B). These findings indicate that Ext1 loss causes baseline ultrastructural glycocalyx depletion, predisposing the lung microvasculature to inflammation‐induced injury.

**FIGURE 7:**
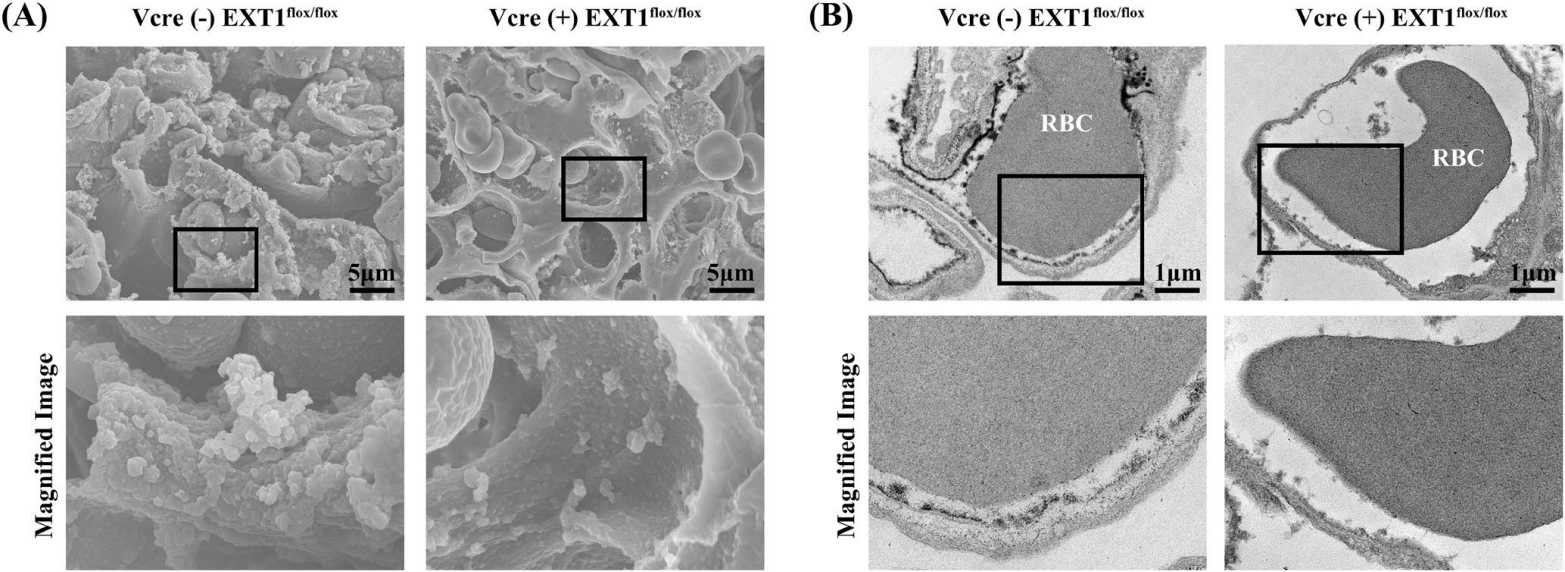
Ultrastructural loss of endothelial glycocalyx in Ext1‐deficient mice revealed by electron microscopy. (A) Representative scanning electron microscopy images of pulmonary capillary luminal surfaces in VE‐cadherin‐Cre (−) Ext1^flox/flox^ and VE‐cadherin‐Cre (+) Ext1^flox/flox^ mice. Control mice display abundant, fine, filamentous glycocalyx structures, whereas Cre^+^ mice show marked thinning and disruption. Magnified views (bottom) highlight the severe loss of glycocalyx in Cre^+^ mice. (B) Transmission electron microscopy images of pulmonary capillaries showing the endothelial surface layer (black box) adjacent to red blood cells (RBCs). A continuous electron‐dense glycocalyx layer is present in control mice. Ext1 deficiency results in a severely attenuated or absent glycocalyx, confirming structural destruction at the ultrastructural level. Magnified images (bottom) emphasize the loss of the surface layer in Cre^+^ capillaries.

### 3.4 Endothelial Ext1 Deficiency Prolongs Pulmonary Inflammation and Alters Immune Cell Dynamics Following Endotoxin Challenge

To examine the role of endothelial Ext1 during inflammatory stress, VE‐cadherin‐Cre; Ext1^flox/flox^ mice and control littermates were challenged with LPS. Proinflammatory cytokine interleukin‐6 (IL‐6) serum levels increased rapidly after LPS administration in both groups, but the peak response at 6 h was significantly attenuated in Ext1‐deficient mice compared with that for controls (Figure 8A). IL‐6 concentrations subsequently declined in both groups, returning to baseline by 48 h.

**FIGURE 8:**
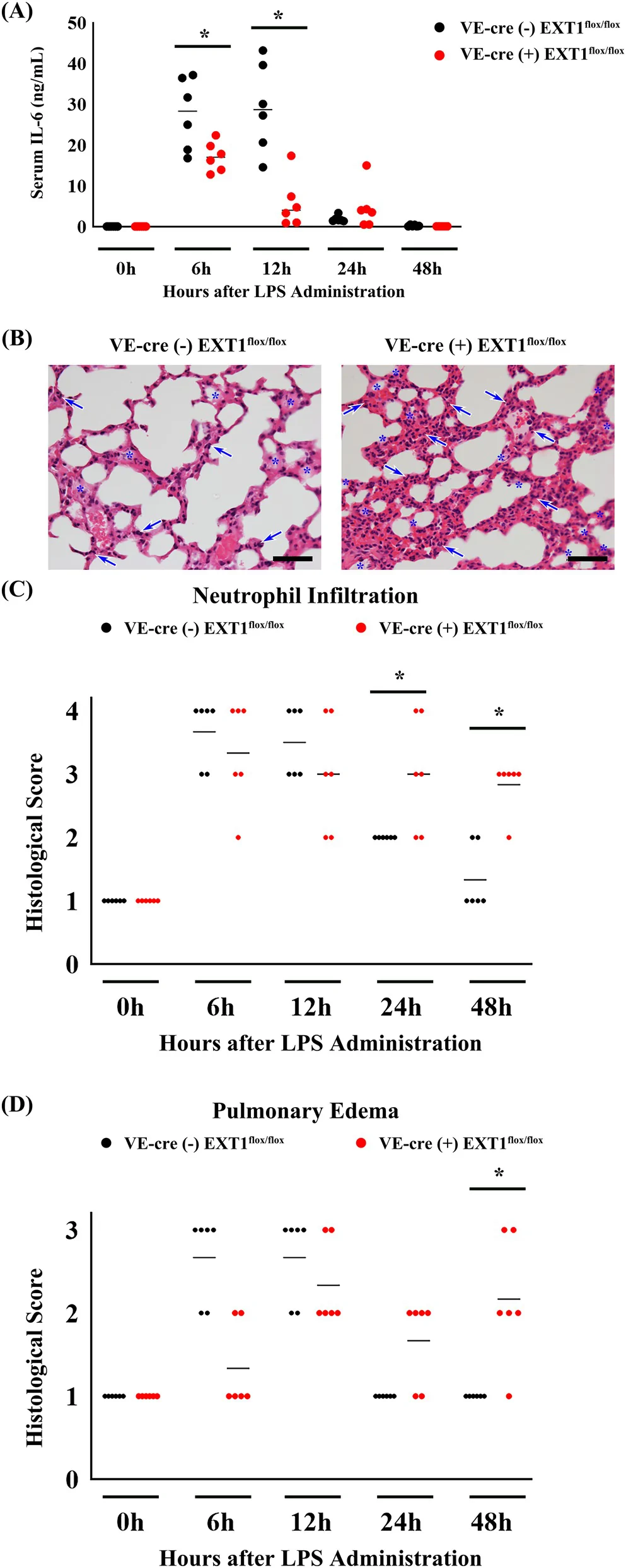
Altered cytokine kinetics and exacerbated lung inflammation in endothelial Ext1‐deficient mice following lipopolysaccharide (LPS) administration. (A) Plasma interleukin (IL)‐6 concentrations at the indicated time points after intraperitoneal LPS injection. VE‐cadherin‐Cre(+) Ext1flox/flox mice exhibited lower circulating IL‐6 concentrations despite more severe lung injury. (B) Representative hematoxylin and eosin (H&E)‐stained lung sections obtained 48 h after LPS administration. Arrows indicate inflammatory cell infiltration, and asterisks indicate areas of pulmonary edema. (C) Histological scores for neutrophil infiltration at the indicated time points after LPS administration. (D) Histological scores for pulmonary edema at the indicated time points after LPS administration. Data are presented as individual values with the group means. Statistical significance was assessed using Student's two‐tailed *t*‐test for panel A and the Mann–Whitney U test for panels C and D. *p < 0.05 versus Cre^−^ controls.

Following LPS administration, Ext1‐deficient mice exhibited lower circulating IL‐6 levels despite more severe and prolonged lung inflammation.

Although circulating IL‐6 levels were reduced, the biological significance of this finding remains uncertain. Reduced plasma IL‐6 concentrations do not necessarily indicate attenuation of inflammatory signaling, and the mechanisms underlying this observation remain to be determined.

Consistent with this interpretation, Ext1‐deficient mice displayed persistent neutrophil infiltration, delayed clearance of CD11b^+^ myeloid cells, sustained macrophage accumulation, and prolonged pulmonary edema, indicating unresolved tissue‐level inflammation.

Histological examination of lung tissues revealed typical acute inflammatory changes after LPS stimulation, characterized by alveolar wall thickening, interstitial cell infiltration, and occasional intra‐alveolar exudates (Figure 8B). Although both groups exhibited early neutrophil infiltration, Ext1‐deficient mice showed delayed resolution, with persistently elevated histological scores for neutrophil infiltration and pulmonary edema at 24 and 48 h (Figure 8C).

To further elucidate the mechanisms underlying the prolonged inflammation in Ext1‐deficient mice, we next examined inflammatory cell recruitment using immunohistochemistry. CD11b‐positive cells, representing infiltrating myeloid cells, accumulated rapidly after LPS challenge in both groups, peaking at 12 h (Figure 9A). However, the number of CD11b‐positive cells declined earlier in controls, whereas it remained elevated in Ext1‐deficient mice at 48 h (Figure 9B), indicating delayed inflammatory cell clearance. Conversely, F4/80‐positive macrophages gradually increased over time and were more abundant in Ext1‐deficient mice at 48 h (Figure 9C,D). These results suggest that loss of endothelial Ext1 alters the amplitude of cytokine responses and disrupts the orchestration of immune cell recruitment and clearance, prolonging pulmonary inflammation after endotoxin challenge.

**FIGURE 9:**
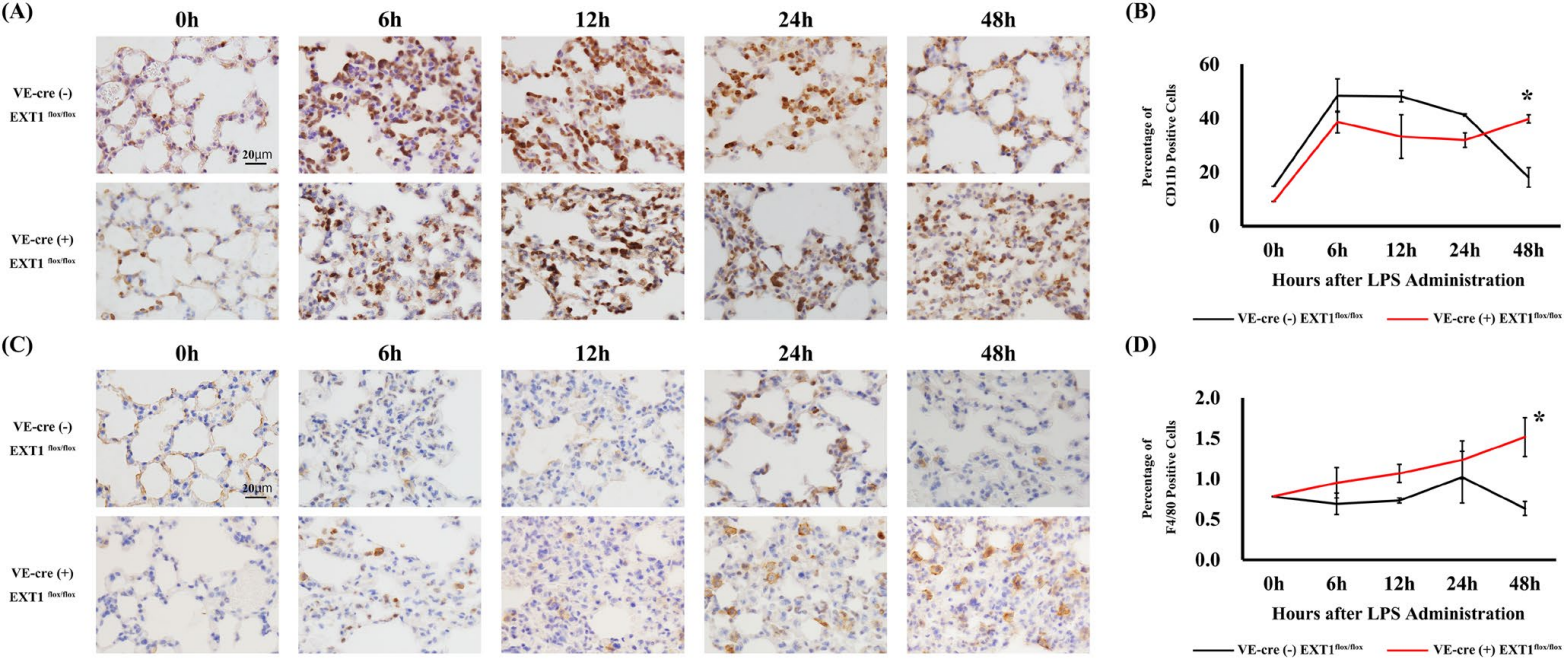
Prolonged inflammatory cell accumulation in Ext1‐deficient mice during lipopolysaccharide (LPS)‐induced lung injury. (A) Immunohistochemical staining for CD11b^+^ cells at 0–48 h after LPS administration. (B) Quantification of CD11b^+^ cells. Ext1‐deficient mice (red line) show higher CD11b^+^ cell retention at 48 h, indicating delayed inflammatory resolution. (C) Immunohistochemical staining for F4/80^+^ macrophages. (D) Quantification of F4/80^+^ cells. Cre^+^ Ext1^flox/flox^ mice show persistent and increased macrophage accumulation, consistent with impaired inflammatory clearance. Data represent mean ± standard error of the mean (SEM). *p < 0.05 vs. Cre^−^ controls.

### 3.5 Endothelial Ext1 Deficiency Reprograms the Endothelial Transcriptome Toward Immune Activation

To further explore the molecular mechanisms underlying the prolonged inflammatory phenotype observed in Ext1‐deficient mice, transcriptomic profiling was performed using endothelial cells isolated from the lungs of VE‐cadherin‐Cre; Ext1^flox/flox^ and control mice. Gene set enrichment analysis revealed that multiple immune‐related pathways were significantly upregulated in Ext1‐deficient endothelial cells compared with controls. Among the top enriched pathways were leukocyte extravasation signaling (Figure 10A), natural killer cell‐mediated cytotoxicity (Figure 10B), and chemokine signaling (Figure 10C), indicating that endothelial Ext1 loss promotes a pro‐inflammatory and leukocyte‐interactive phenotype. Furthermore, the vascular endothelial growth factor (VEGF) A signaling pathway was enriched (Figure 10D), suggesting dysregulated vascular signaling and altered endothelial barrier homeostasis. These transcriptional changes mirror the persistent inflammation and enhanced leukocyte infiltration observed in vivo (Figures 8 and 9), and collectively demonstrate that loss of endothelial Ext1 triggers a cell‐autonomous shift in endothelial gene expression toward immune activation and inflammatory signaling.

**FIGURE 10:**
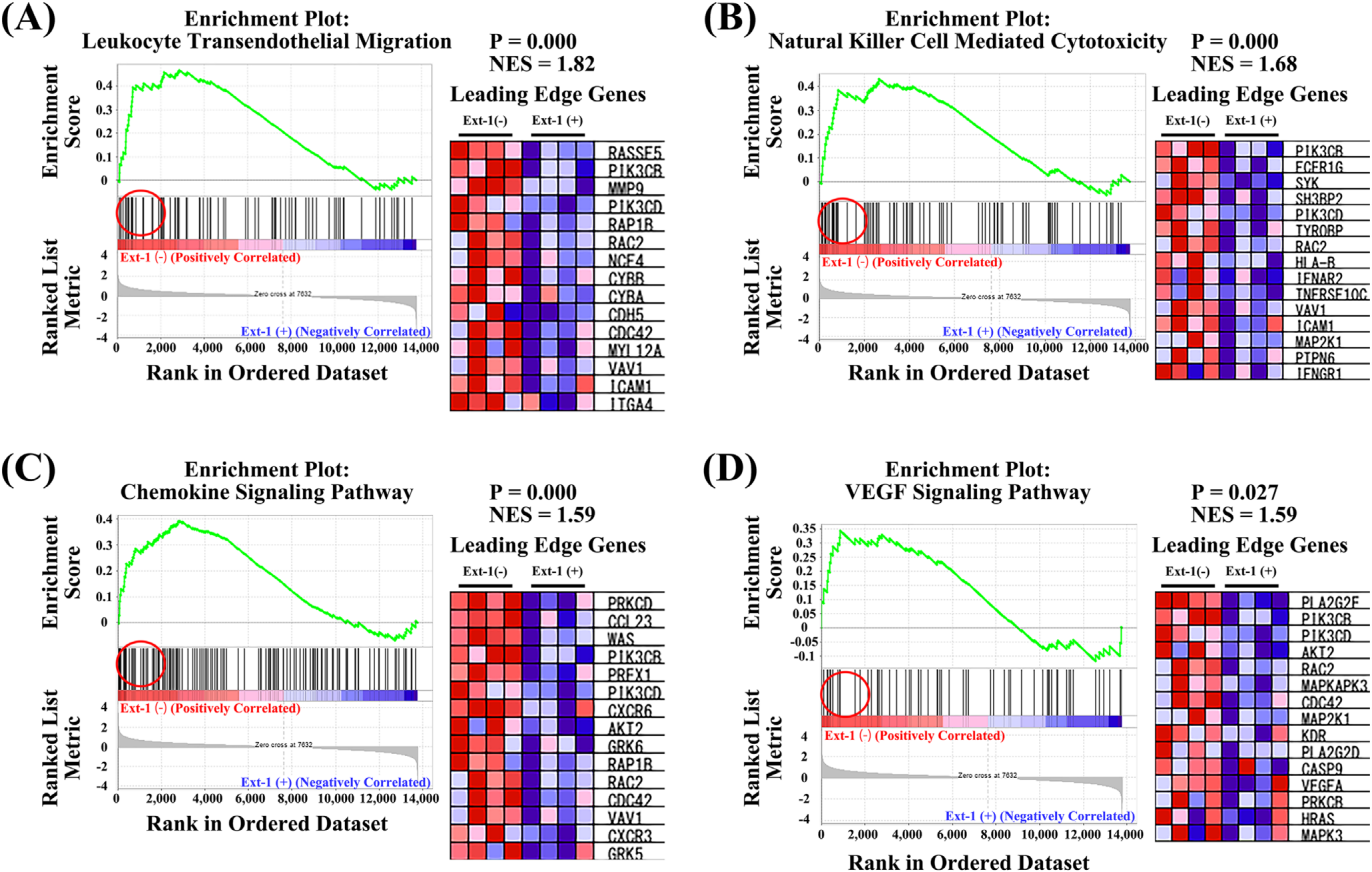
Gene set enrichment analysis revealing immune activation and chemokine signaling in Ext1‐deficient endothelial cells. (A) Enrichment plot for leukocyte transendothelial migration. (B) Natural killer cell‐mediated cytotoxicity. (C) Chemokine signaling pathway. (D) Vascular endothelial growth factor (VEGF) signaling pathway. Heatmaps (right of each plot) show leading‐edge genes driving the enrichment signal.

## 4 Discussion

The eGCX is increasingly recognized as a critical regulator of vascular homeostasis, mechanotransduction, and inflammation. Its major glycosaminoglycan component, HS, requires Ext1‐mediated chain elongation for proper synthesis. Systemic conditions such as diabetes, aging, chronic kidney disease, and sepsis cause glycocalyx degradation and are associated with prolonged or dysregulated inflammation. These data indicate that endothelial Ext1 is indispensable for maintaining eGCX integrity, preserving endothelial transcriptional homeostasis, and supporting efficient inflammatory resolution during LPS‐induced lung injury.

### 4.1 Ext1 Is Essential for eGCX Structural Maintenance

SEM and TEM revealed that Ext1‐deficient endothelial cells lacked the luminal glycocalyx even at baseline, consistent with reduced transcriptomic expression in HS‐biosynthetic enzymes and core proteoglycans. The endothelial surface flattening was similar to pathological changes previously reported in heparanase activation, ischemia–reperfusion injury, and septic shock [39, 40, 41, 42], supporting that Ext1‐mediated HS synthesis is indispensable for eGCX formation and stability.

### 4.2 Despite Lower Circulating IL‐6, Ext1 Deficiency Prolongs Inflammation and Worsens Lung Injury

Although plasma IL‐6 levels were lower in Ext1‐deficient mice after LPS challenge, all histological and cellular endpoints, including neutrophil infiltration, CD11b^+^ and F4/80^+^ cell accumulation, pulmonary edema, and tissue injury, were worse and more persistent. This apparent dissociation suggests that circulating cytokine concentrations may not necessarily reflect the magnitude of local tissue inflammation in the setting of endothelial glycocalyx disruption. Because cytokine levels within affected organs were not assessed in the present study, the mechanisms underlying the reduced plasma IL‐6 concentrations remain uncertain. One possible explanation is that altered endothelial barrier function and vascular compartmentalization may influence the distribution of inflammatory mediators. This interpretation is consistent with previous reports suggesting that severe glycocalyx disruption can promote cytokine extravasation and alter measurable systemic cytokine levels [11, 43]. Further studies examining cytokine expression within affected organs will be required to clarify this relationship.

The increased accumulation of CD11b^+^ and F4/80^+^ cells observed in Ext1‐deficient lungs further supports the presence of persistent inflammation during the late phase of injury. However, CD11b and F4/80 expression alone do not permit definitive characterization of macrophage polarization status. Therefore, the functional phenotype of these cells remains uncertain, and our data cannot distinguish between persistence of pro‐inflammatory macrophages, expansion of resolution‐associated macrophages, or impaired transition between these phenotypic states. Further studies incorporating macrophage polarization markers and cytokine profiling will be required to clarify the role of these cells in glycocalyx‐dependent inflammatory resolution.

Collectively, these findings suggest that Ext1‐dependent HS is important not only for maintaining endothelial barrier integrity but also for coordinating appropriate inflammatory responses and their resolution.

### 4.3 Ext1 Loss Reprograms Endothelial Transcription

RNA‐seq revealed an immune‐activated but structurally compromised transcriptional profile in Ext1‐deficient endothelial cells. Immune‐related pathways were upregulated, while extracellular matrix, adhesion, and angiogenic programs were downregulated. This dual phenotype suggests heightened inflammatory sensitivity combined with impaired regenerative capacity, consistent with HS functioning as a cofactor for growth factors such as VEGF and fibroblast growth factor [44, 45, 46].

A notable strength of the present study is the use of an endothelial‐specific Ext1‐deficient model, which allowed us to examine the consequences of constitutive heparan sulfate deficiency independent of acute glycocalyx shedding. Previous studies have largely focused on enzymatic degradation of the glycocalyx during sepsis, ischemia–reperfusion injury, or inflammatory stress. In contrast, our findings demonstrate that impaired heparan sulfate biosynthesis alone is sufficient to establish a chronically vulnerable endothelial phenotype characterized by increased permeability, altered inflammatory responses, and delayed resolution of tissue injury. These observations support the concept that the endothelial glycocalyx is not merely a passive barrier but an active regulator of inflammatory homeostasis.

### 4.4 Glycocalyx Injury as a Unifying Mechanism in Diabetes, Endothelial Dysfunction, and Infectious Disease Severity

Our findings provide mechanistic insight into why chronic endothelial dysfunction, particularly diabetes, predisposes individuals to prolonged or dysregulated inflammation. Diabetes reduces Ext1 expression, disrupts HS structure, and damages the glycocalyx through hyperglycemia, oxidative stress, and low‐grade inflammation [47, 48, 49]. These data support glycocalyx degradation as a contributor to delayed inflammatory resolution.

Many comorbidities associated with severe infectious diseases (aging, chronic kidney disease, hypertension, dyslipidemia, and metabolic syndrome) share this phenotype of glycocalyx vulnerability. Clinical evidence shows that coronavirus disease 2019 severity correlates strongly with glycocalyx shedding, reflected by elevated SDC‐1 and HS fragments [50, 51]. Severe acute respiratory syndrome coronavirus 2 can utilize HS as a cofactor for cellular entry [52] and promote heparanase activation [53], further linking glycocalyx integrity to host response. Ext1‐dependent HS biosynthesis may represent a common mechanistic pathway explaining why individuals with underlying vascular disease experience excessive tissue injury and impaired inflammatory resolution during infections.

### 4.5 Clinical Implications

These findings highlight the therapeutic potential of strategies aimed at stabilizing or restoring the endothelial glycocalyx, including HS mimetics, antithrombin, sphingosine‐1‐phosphate receptor agonists, recombinant plasma gelsolin, and other endothelial‐protective approaches. Measurement of glycocalyx injury markers (e.g., SDC‐1 and HS fragments) may help identify patients at high risk of prolonged inflammation and organ injury. Strategies that preserve or augment Ext1‐dependent heparan sulfate biosynthesis may therefore represent a promising therapeutic approach for acute respiratory distress syndrome (ARDS), severe pneumonia, sepsis, and future pandemics involving endothelial injury.

Although the present study focused on the consequences of endothelial Ext1 deletion rather than its upstream regulation, inflammatory stress and metabolic conditions such as hyperglycemia may influence glycocalyx turnover and HS metabolism.

Although endothelial Ext1 deletion was not directly confirmed at the protein level, transcriptomic analyses together with heparan sulfate staining, ultrastructural findings, and functional abnormalities consistently supported effective endothelial Ext1 deficiency. Reliable protein‐level validation of endothelial glycocalyx‐associated Ext1 would require endothelial cell isolation, a procedure that disrupts the fragile endothelial glycocalyx and therefore does not faithfully preserve the in vivo endothelial phenotype.

Furthermore, while VE‐cadherin‐Cre is widely used for endothelial‐specific gene targeting, our endothelial cell isolation and transcriptomic analyses indicate that the observed phenotype primarily reflects endothelial cell–intrinsic Ext1 deficiency.

## 5 Conclusion

Endothelial Ext1 is indispensable for heparan sulfate synthesis, eGCX structural integrity, and coordinated inflammatory resolution during acute lung injury. Its loss results in glycocalyx destruction, increased vascular permeability, altered systemic cytokine responses, impaired inflammatory resolution, and exacerbated tissue injury. Transcriptomic reprogramming of Ext1‐deficient endothelial cells highlights the central role of HS in maintaining vascular homeostasis. These findings identify Ext1‐dependent glycocalyx preservation as a key determinant of host inflammatory responses and suggest that targeting glycocalyx integrity may represent a promising therapeutic strategy for inflammatory lung diseases, including sepsis, ARDS, and conditions associated with endothelial vulnerability such as diabetes.

## Author Contributions

Yuki Kawasaki: writing – original draft, Investigation, Conceptualization. Hiroyuki Tomita: writing – review and editing, Methodology, Investigation, Funding acquisition, Conceptualization. Kodai Suzuki: Methodology, Investigation, Formal analysis. Chihiro Takada: Methodology, Investigation. Hirotaka Asano: Methodology, Investigation. Soichiro Kano: Methodology, Investigation. Yugo Wakayama: Methodology, Investigation. Hirotsugu Fukuda: Visualization, Investigation. Toru Minamiyama: Methodology, Investigation. Genki Yoshimura: Investigation. Ryo Kamidani: Investigation. Yoshinori Kakino: Investigation, Funding acquisition. Tomotaka Miura: Investigation, Data curation. Yuichiro Kitagawa: Visualization, Funding acquisition. Tetsuya Fukuta: Visualization, Funding acquisition. Takahito Miyake: Visualization, Methodology, Investigation. Akio Suzuki: writing – review and editing. Kazu Matsumoto: writing – review and editing, Supervision. Nobuyuki Tetsuka: Supervision, Methodology. Shozo Yoshida: Supervision, Funding acquisition. Yoichi Maekawa: Methodology. Yu Yamaguchi: writing – review and editing, Supervision, Funding acquisition. Hideshi Okada: writing – review and editing, Supervision, Conceptualization, Funding acquisition. All authors were involved in drafting and revising the manuscript.

## Funding

This work was supported by MEXT; Japan Society for the Promotion of Science (JSPS), grants 20K07587, 20K17856, 21K16570, 22H03171, 23H03326, 23K15612, 23K18311, 24K02545, 24K12174, 24K19484, 25K02742, 25K12267, and 25K19629; the JST FOREST PROGRAM, grant JPMJFR220W; and the Foundation for the National Institutes of Health (FNIH), grant 1R01AG072468.

## Conflicts of Interest

The authors declare no conflicts of interest.

## Data Availability

The RNA sequencing data generated in this study have been deposited in the NCBI Gene Expression Omnibus (GEO) database under BioProject accession number PRJNA1367933. Microarray data have been deposited in GEO under accession number GSE313973. Other data supporting the findings of this study are available from the corresponding author upon reasonable request.
